# Embryonic Pig Pancreatic Tissue Transplantation for the Treatment of Diabetes

**DOI:** 10.1371/journal.pmed.0030215

**Published:** 2006-06-20

**Authors:** Smadar Eventov-Friedman, Dalit Tchorsh, Helena Katchman, Elias Shezen, Anna Aronovich, Gil Hecht, Benjamin Dekel, Gideon Rechavi, Bruce R Blazar, Ilan Feine, Orna Tal, Enrique Freud, Yair Reisner

**Affiliations:** 1Department of Immunology, Weizmann Institute of Science, Rehovot, Israel; **2**Pediatric Hemato-Oncology and Functional Genomics Unit, Sheba Medical Center, Tel Hashomer, Israel; **3**University of Minnesota Cancer Center and Department of Pediatrics, Division of Bone Marrow Transplantation, Minneapolis, Minnesota, United States of America; **4**Department of Pediatric Surgery, Schneider Children's Medical Center of Israel, Petach Tikvah, Israel; United States Public Health Service/National Institutes of Health National Institute of Diabetes and Digestive and Kidney DiseasesUnited States of America

## Abstract

**Background:**

Transplantation of embryonic pig pancreatic tissue as a source of insulin has been suggested for the cure of diabetes. However, previous limited clinical trials failed in their attempts to treat diabetic patients by transplantation of advanced gestational age porcine embryonic pancreas. In the present study we examined growth potential, functionality, and immunogenicity of pig embryonic pancreatic tissue harvested at different gestational ages.

**Methods and Findings:**

Implantation of embryonic pig pancreatic tissues of different gestational ages in SCID mice reveals that embryonic day 42 (E42) pig pancreas can enable a massive growth of pig islets for prolonged periods and restore normoglycemia in diabetic mice. Furthermore, both direct and indirect T cell rejection responses to the xenogeneic tissue demonstrated that E42 tissue, in comparison to E56 or later embryonic tissues, exhibits markedly reduced immunogenicity. Finally, fully immunocompetent diabetic mice grafted with the E42 pig pancreatic tissue and treated with an immunosuppression protocol comprising CTLA4-Ig and anti–CD40 ligand (anti-CD40L) attained normal blood glucose levels, eliminating the need for insulin.

**Conclusions:**

These results emphasize the importance of selecting embryonic tissue of the correct gestational age for optimal growth and function and for reduced immunogenicity, and provide a proof of principle for the therapeutic potential of E42 embryonic pig pancreatic tissue transplantation in diabetes.

## Editors' Summary

**Background.:**

Diabetes is a growing global health problem. By 2030, more than 300 million people around the world will have this chronic, incurable disorder, double the current number. In non-diabetic people, cells in the pancreas called beta cells release insulin, a hormone that controls the level of sugar (glucose) in the blood. In diabetics, blood-sugar levels become dangerously high either because the beta cells have been destroyed so no insulin is made (type 1 diabetes, 5%–10% of all cases) or because the cells that normally remove sugar from the blood have become insensitive to insulin (type 2 diabetes). In particularly severe cases of type 2 diabetes, the beta cells also stop releasing insulin. People with type 2 diabetes can usually control their blood-sugar levels through diet and exercise and by taking oral anti-diabetic drugs; people with type 1 diabetes or severe type 2 diabetes have to replace the missing insulin by injections. It is very important that diabetics keep their blood-sugar levels as normal as possible to minimize the disorder's serious long-term complications. These include kidney failure, blindness, nerve damage, and an increased risk of heart disease and strokes.

**Why Was This Study Done?:**

While individuals with type 1 diabetes can control their blood-sugar levels pretty well by carefully monitoring their life style and injecting insulin, potentially better control and fewer long-term complications can be achieved by providing a new source of insulin-producing cells through transplantation of pancreatic tissue from a dead human donor. However, because there is not enough human pancreatic tissue to treat all the diabetics who could benefit from such transplants, researchers are investigating other sources of insulin-producing cells. One possibility is pig pancreatic tissue. Glucose control is very similar in pigs and humans, pig insulin injections have been used for years to control diabetes, and pigs are in plentiful supply. However, besides general concerns about xenotransplantation (that is, transplantation from a foreign species such as pigs into humans), early attempts to treat human diabetes by transplantation of pancreatic tissue taken from pig embryos at late stages of gestation were not successful. The researchers involved in this study had done earlier experiments that suggested that the age of the pig donor tissue influences how well transplantation into other species works. They therefore wanted to test whether pancreatic tissue from younger pig embryos might work better for pancreas transplants: they hoped that younger tissue would grow and integrate better with the surrounding host tissue. Additionally, a major concern with all transplantations is whether the transplanted cells or tissue will be recognized as foreign and as such destroyed by the host's immune system. Because tissue from younger embryos is generally less likely to trigger an immune reaction, the researchers hoped that pancreatic tissue from younger pig embryos would be less readily recognized as foreign by the human immune system.

**What Did the Researchers Do and Find?:**

They started by transplanting pancreatic tissue from pig embryos of different ages into mice with defective immune systems. Tissue taken about a third of the way through gestation (that is, from embryos 42 or 56 days old) grew better than tissue taken earlier or later, secreted more pig insulin over extended periods of time, and was better at maintaining normal blood-sugar levels when the beta cells of the host mice were destroyed. The researchers then examined whether embryonic pig pancreatic tissue of different ages triggered an immune reaction by seeing how well it survived when human immune system cells were also transplanted into the mice. Tissue from 42-day-old embryos came out best in this test too, suggesting that there is little or no “direct” immune reaction by circulating immune cells against pancreatic tissue from this stage. Finally, the researchers transplanted pancreatic tissue of this age into diabetic mice with an intact immune system. These mice rejected the transplants (presumably through an “indirect” immune reaction), but that rejection could be overcome when the recipient mice were treated with drugs that suppressed the part of their immune system that is responsible for these indirect immune reactions. (Human patients who receive a transplant are usually treated with drugs that suppress direct and indirect immune reactions.) When the mice were kept on the drugs, the grafts survived in the long term, and the mice had normal blood-sugar levels once the graft was well established.

**What Do These Findings Mean?:**

These results suggest that the exact age of embryonic pig pancreatic tissue influences how well the transplanted tissue grows and integrates into a host from a different species (in this case, the mouse) and how strong an immune reaction it triggers. Overall, these results support the notion that pig embryonic pancreas tissue could potentially be a source of tissue for transplantation into human patients with diabetes. The next steps in exploring this possibility are likely to involve experiments in monkeys to find out how much tissue should be implanted and where, and to check that the transplanted tissue remains functional in these animals. The ability of the 42-day-old embryonic tissue to avoid direct immune rejection also needs to be confirmed. And, ideally, the goal remains to find ways to avoid an immune reaction altogether, so that recipients of transplants do not need to be continually treated with drugs that suppress their immune system (which makes them more susceptible to infections and can have other side effects). Xenotransplantation has potential benefits and risks and remains controversial. Studies like this one and others that seek to better understand the risks and benefits are necessary to allow reasonable decisions to be made.

**Additional Information.:**

Please access these Web sites via the online version of this summary at http://dx.doi.org/10.1371/journal.pmed.0030215

• MedlinePlus pages on diabetes and on pancreas transplantation

• Information from the Juvenile Diabetes Research Foundation International Description

• Wikipedia pages on diabetes, xenotransplantation, and pancreas transplantation (note: Wikipedia is a free online encyclopedia that anyone can edit)

## Abstract 3

Pancreatic tissue from embryonic pigs co-transplanted with or without human immune cells into immune-deficient mice suggests that the embryonic stage of the pig donor affects the immunogenicity of the graft.

## Introduction

Diabetes mellitus is a severe and debilitating chronic disease that develops in nearly 5% of the world's population [ 1, 2]. For all patients who have type 1 diabetes (insulin deficiency due to autoimmune destruction of beta cells) or severe type 2 diabetes (impaired insulin secretion combined with reduced sensitivity to insulin), the only practical treatment possible today is lifelong insulin replacement by multiple daily injections. However, even for very compliant patients participating in a carefully titrated insulin therapy protocol aimed at avoiding glycemic fluctuations, it is difficult to tightly control blood glucose levels. Thus, severe microvascular complications associated with the disease may develop [ 3– 6]. Transplantation of an entire pancreas or pancreatic islets would potentially benefit millions of such patients [ 7– 13]. Unfortunately, this alternative is extremely limited by the shortage of human donor organs available for transplantation [ 14– 16].

Considering the ethical limitations associated with implementation of new strategies for islet allotransplantation, such as from human embryonic tissues, and the ongoing debate regarding the use of human embryonic stem cells or adult pancreatic stem cells for transplantation, the use of porcine tissues might potentially provide an attractive, unlimited source of pancreatic tissue [ 17].

Several safety concerns, in particular the potential hazards associated with endogenous porcine retroviruses, have presented major obstacles to such xenotransplantation [ 18– 20]. However, it is important to note that previous pig-to-human xenotransplantations have not revealed a single instance of porcine retrovirus transmission to a human [ 21]. Moreover, a recent study has suggested that these viruses could be eradicated from pig herds bred specifically for xenotransplantation [ 22]. Thus, as argued recently by Ogata and Platt [ 23], although the potential threat of porcine retroviruses to public health cannot be entirely dismissed, it should be approached by careful attention to the xenograft recipients, rather than being a reason for abandoning the xenotransplantation approach.

A second major challenge, which still represents a major obstacle for xenotransplantation, is the immune barrier [ 23]. However, the reduced immunogenicity of embryonic tissues [ 24– 26] might be advantageous in ameliorating rejection following implantation of pig embryonic pancreas.

In the present study, we evaluated in the NOD-SCID mouse model the ability of embryonic pig pancreatic tissue obtained at different gestational time points to grow and secrete insulin for prolonged time periods, and we further tested the grafts' ability to normalize blood glucose levels in diabetic mice. An optimal “window of opportunity” was defined by measuring both the growth potential of the graft and its ability to avoid graft rejection. The latter was evaluated by using models for both the direct and indirect rejection pathways.

## Methods

### Animals

Animals were maintained under conditions approved by the Institutional Animal Care and Use Committee at the Weizmann Institute of Science. Immune-deficient NOD-SCID mice aged 8–10 wk were used as hosts for the studies of graft growth, and C57BL/6 mice were used as immunocompetent mice for rejection studies; all mice were obtained from the Weizmann Institute Animal Breeding Center (Rehovot, Israel). For evaluation of rejection pathways, CBA/CaHN-Btk XID mice and CD1-Hfh11 nude mice were purchased from the Roscoe B. Jackson Memorial Laboratory (Bar Harbor, Maine, United States). All mice were maintained in small cages (up to five animals in each cage) and fed sterile food and acid water containing ciprofloxacin (20 μg/ml).

### Porcine Embryonic Pancreatic Tissues

Pig embryos were obtained from the Lahav Institute of Animal Research (Kibbutz Lahav, Israel). Pregnant sows were operated on at precisely defined stages of their pregnancy (embryonic day 24 [E24], E28, E42, E56, E80, and E100) under general anesthesia, and embryos were extracted. Warm ischemia time was less than 10 min, and the embryos were transferred in cold phosphate buffered saline. Pig pancreas precursors for transplantation were extracted under a light microscope and maintained under sterile conditions at 4 °C in RPMI 1640 (Biological Industries, Beit Haemek, Israel) pending transplantation. Cold ischemia time until transplantation was less than 2 h. The study protocol was approved by the ethics committees at both the Weizmann Institute of Science and the Lahav Institute of Animal Research.

### Transplantation Procedure

Implantation of pig embryonic pancreas was performed under general anesthesia with 2,2,2-tribromoethanol 97% (2.5% Avertin in phosphate buffered saline; Sigman-Aldrich; http://www.sigmaaldrich.com) at a dose of 10 ml/kg injected intraperitoneally. Host kidney was exposed through a left lateral incision. A 1.5-mm incision was made at the caudal end of the kidney capsule, and the whole organ or a single fragment (depending on gestational age) of the pancreatic tissue, 1–2 mm in diameter, was grafted. Each group studied included 5–7 grafted mice with donor tissue of distinct gestational age exposed to various experimental conditions.

### Glucose Challenge Test

Mice fasted 10–12 h were injected intraperitoneally with glucose (3 g/kg) 6 wk after transplantation. Blood was withdrawn from the retro-orbital sinus before ( *T*_0_) and 30 min after ( *T*_30_) glucose administration, and tested for both glucose (Accu-check glucose sticks; Roche; http://www.roche.com/home.html) and pig insulin levels (measured by ELISA). In diabetic NOD-SCID mice the test continued for 150 min.

### Induction of Diabetes

To determine the capacity of the pig embryonic pancreatic grafts to regulate hyperglycemia in mice, grafted (E42 pig embryonic grafts 4 mo after grafting) and non-grafted immune-deficient NOD-SCID or C57BL/6 mice were intravenously injected with 90 mg/kg alloxan (Sigma-Aldrich). Glucose levels were measured from blood collected from the tip of the tail following alloxan treatment at different time points. In a second model, intact NOD-SCID mice were injected intraperitoneally with 35 mg/kg/d streptozotocin solubilized in sodium citrate buffer (pH 4.5) (Sigma-Aldrich) daily for 5 d. Transplantation of E42 pancreas was carried out 3 d after completing the streptozotocin injections, and only in animals with a glucose level above 16.5 mmol/l on two consecutive days prior to transplantation. Daily insulin (Lantus, Sanofi-Aventis, Paris, France) was given intraperitoneally between 8:00 and 10:00 a.m. Blood glucose was measured twice weekly at noon time, while postponing the daily insulin injections, and if the glucose level was less than 13.7 mmol/l on two consecutive tests, insulin administration was withheld.

### Isolation and Transfer of Human Peripheral Blood Mononuclear Cells

Human peripheral blood mononuclear cells (hu-PBMCs) were generated from buffy coats obtained from normal volunteers, layered on Ficoll-Paque solution, and centrifuged at 2,000 rpm for 20 min. Human cells (80 × 10 ^6^), collected from the interface layer, were injected intraperitoneally after washing to NOD-SCID mice, 1–3 d following embryonic pancreatic transplantation. Engraftment of hu-PBMCs was analyzed by measuring human immunoglobin G (IgG) levels in the mouse serum by ELISA, and by detection of CD45 human leukocytes in the peritoneal fluid, 10–14 d after infusion, by a FACS analyzer (Becton-Dickinson; http://www.bd.com) using anti-human-leukocyte common antigen CD45/FITC (clone T29/33; Dako; http://www.dako.com). Only mice positive by ELISA for human IgG production or by FACS for human immune cell engraftment were further analyzed.

### Costimulatory Molecular Blockade

Following transplantation, grafted C57BL/6 mice were treated with mouse CTLA4-Ig fusion protein (lot number 20204, Chimerigen Laboratories; http://www.chimerigen.com) and anti-CD40L antibody (MR1, kindly provided by Prof. Bruce Blazar, University of Minnesota, Minneapolis, Minnesota, United States) given intraperitoneally on days 0, 2, 4, and 6 at doses of 200 μg/mouse (CTLA4-Ig) and 250 μg/mouse (anti-CD40L). Single injections of CTLA4-Ig and anti-CD40L were repeated every 2 wk. Control mice were injected with phosphate buffered saline.

### Histology and Immunohistochemistry

Tissue sections were routinely stained by Hematoxylin and Eosin. Assessment of graft differentiation and function was performed by histochemical and immunohistochemical labeling of 4-μm-thick paraffin sections after xylene deparaffinization and rehydration. Endogenous peroxidase, in both paraffin sections and 6- or 12-μm cryosections, was blocked with 0.3% H _2_O _2_ in 70% methanol for 10 min. Antigen retrieval procedures were performed according to the manufacturer's instructions for each antibody (see below). After blocking, sections were incubated with specific first antibody for 1 h at room temperature. The following antibodies were used: rabbit anti-human glucagon (Dako), guinea pig anti-rabbit insulin (Dako), rabbit anti-human pancreatic polypeptide (Dako), a cocktail of rabbit anti-porcine α-amylase and bovine trypsin (Nordic Immunology, Tilburg, Netherlands), mouse anti-human Ki67 (clone MIB-1; Dako), mouse anti-human cytokeratin 20 (clone Ks 20.8; Dako), mouse anti-human CD45 (clones 2B11 and PD7/26; Dako), polyclonal rabbit anti-human CD3 (Dako), mouse anti-human CD20cy (clone L26; Dako), mouse anti-human CD68 (clone KP1; Zymed Laboratories, San Francisco, California, United States), mouse anti-pig CD45 (MCA1447; Serotec, Oxford, United Kingdom), and rat anti-mouse F4/80 antigen (Serotec). Detection of antibody binding was performed using the following secondary reagents: Dako peroxidase envision system for detection of mouse and rabbit antibodies and Sigma biotinylated anti-goat antibody (followed by extra-avidin peroxidase reagent) for detection of goat antibodies. For all peroxidase-labeled sections, diaminobenzidine was used as the chromogen. Tissue sections were counterstained with Hematoxylin.

For triple immunofluorescent labeling with first antibodies including mouse anti-human Ki67, guinea pig anti-rabbit insulin, and a cocktail of rabbit anti-porcine α-amylase and bovine trypsin, the following secondary antibodies were applied: Texas Red conjugated donkey anti-mouse antibody (Jackson ImmunoResearch Laboratories, West Grove, Pennsylvania, United States), biotinylated donkey anti–guinea pig antibody following streptavidin aminomethylcoumarin (Jackson ImmunoResearch Laboratories), and Cy2 conjugated goat anti-rabbit antibody (Jackson ImmunoResearch Laboratories). For all immunohistochemical stainings, a negative control was run using the same technique but omitting the primary antibody, while adding the labeled secondary antibody.

### ELISA Measurements of Pig Insulin

The porcine/human insulin kit (K6219, Dako), in which the primary pig anti-insulin antibody does not cross-react with mouse insulin, was used to follow pig insulin levels according to the manufacturer's instructions. Mice serum samples were loaded in a blinded fashion into the wells.

### Statistical Analysis

Differences between groups were evaluated by the Student's *t*-test. Data are expressed as mean ± standard deviation and are considered statistically significant if *p*-values were 0.05 or less.

## Results

### Growth Potential and Functionality of Pig Embryonic Tissue Obtained at Different Gestational Time Points

Earlier studies in SCID mice, in which pig embryonic pancreatic tissue obtained at different gestational ages was implanted and observed for 6 wk, indicated that optimal growth potential is exhibited by the tissue harvested in the E42–E56 period [ 27]. To further ascertain the utility of E42–E56 tissue as a source for transplantation, we initially tested the response of the growing implants in NOD-SCID mice to glucose challenge 6 wk after transplantation. A summary of ten independent experiments is shown in Figure 1; in each experiment, pig pancreatic implants using embryonic tissues of two or more gestational ages were compared. Glucose levels and pig insulin secretion were determined before ( *T*_0_) and 30 min after ( *T*_30_) glucose administration to fasting transplanted mice. Only mice exhibiting a glucose level above 16.5 mmol/l at *T*_30_ were included. Average glucose levels at *T*_0_ and *T*_30_ were 3.5 ± 0.55 and 26.5 ± 3.7 mmol/l, respectively ( *p <* 0.001), in all tested mice. A considerably higher pig insulin level at *T*_30_ compared to *T*_0_ was detected upon glucose stimulation of the mice implanted with E42 or E56 tissue ( *p <* 0.001 and *p <* 0.005, respectively) while glucose stimulation of mice with earlier (E24 and E28) or later (E100) gestational age pancreatic grafts resulted in only minor elevation of pig insulin levels, which was not statistically significant when comparing the *T*_0_ versus the *T*_30_ insulin levels. Although the E80 pig pancreatic grafts demonstrated significant elevation in pig insulin secretion following glucose stimulation ( *T*_0_ versus *T*_30_, *p <* 0.02), the total level of pig insulin was significantly lower than the level secreted by the E42 or E56 implants at *T*_30_ ( *p <* 0.002 and *p <* 0.004, respectively). A significant difference was also noted when comparing the levels of pig insulin secreted at *T*_30_ by the E42 and E56 grafts to those secreted by grafts of earlier gestational ages (E24 and E28) ( *p <* 0.01 and *p <* 0.006, respectively). No significant difference was noted when comparing the *T*_30_ insulin levels secreted by the E42 versus the E56 pancreatic implants.

**Figure 1:**
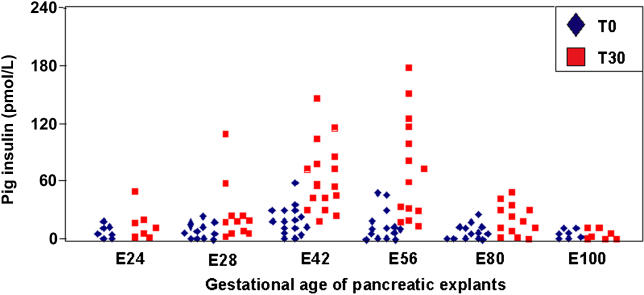
Pig Insulin Secretion by Embryonic Pancreatic Precursors of Different Gestational Ages Transplanted under the Kidney Capsule of NOD-SCID Mice in Response to Glucose Stimulation Mice were tested 6 wk after transplantation, and pig insulin levels were measured before ( *T*_0_) and 30 min after ( *T*_30_) glucose challenge. Each dot represents one mouse. Groups of mice grafted with E24, E28, E42, E56, E80, and E100 donor pancreatic tissues included seven, 11, 16, 14, 13, and seven recipients, respectively.

However, the blood levels of pig insulin in the recipient mice at 6 wk post-transplant were insufficient to control hyperglycemia; therefore, it was of interest to further characterize the gestational “window” by evaluating long-term growth and functionality, as well as the immunogenicity of embryonic tissues harvested at different gestational time points.

Prolonged functional follow-up of the grafted growing tissue when tested at 3 and 4 mo post-transplant revealed an even greater difference when comparing basal serum pig insulin levels secreted by the E42 and E56 grafts with earlier or later gestational age grafts ( Figure 2A). Thus, 12 wk after transplantation, the average pig insulin levels secreted by the E42 and the E56 implants were 85.8 ± 49.8 and 121.2 ± 67.8 pmol/l, respectively, while the E80 implants yielded an average insulin level of only 9.6 ± 6.6 pmol/l ( *p <* 0.001). At 16 wk post-transplant, the E80 implants secreted negligible levels of pig insulin (3 ± 1.2 pmol/l). Although the pig insulin levels secreted by E28, E42, and E56 implants increased over time, significant differences were found when comparing the E28 and the E42 pig insulin levels at 12 and 16 wk after transplantation ( *p <* 0.004 and *p* < 0.003, respectively) and when comparing the pig insulin levels secreted by the E56 and E28 implants at the same time points following transplant ( *p <* 0.007 and *p* < 0.04, respectively), whereas comparison of the pig insulin levels secreted by the E42 and E56 grafts at 6, 12, and 16 wk after transplantation showed no significant difference. Ten months' follow-up of pig insulin blood levels after implantation of E42 tissue revealed a steady state beyond the 4-mo time point (data not shown). The impressive growth of E42 pig pancreatic tissue is shown in Figure 2B, which shows an implant of about 1 mm ^2^ placed under the renal capsule that reached a size of 50 mm ^2^ ( Figure 2B, panel A) 5 mo after transplantation and was composed of normal-looking large islets ( Figure 2B, panel B). The islets were closely adjacent to each other, surrounded by the pancreatic mesenchyme and fat. Typically, most of the islets expressed insulin, glucagon, and pancreatic polypeptide ( Figure 2B, panels C, D, and E, respectively). Epithelial cells within the graft were stained for cytokeratin 20, known for its selective binding to pig pancreas epithelium [ 28], thereby indicating the specific donor origin of this tissue ( Figure 2B, panel F).

**Figure 2:**
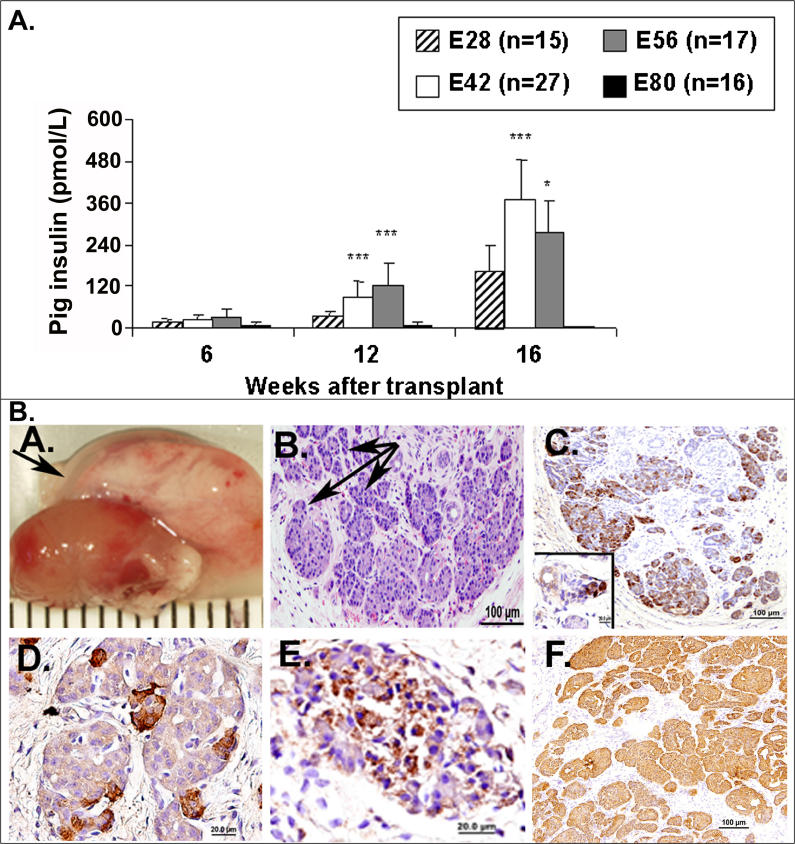
Pig Insulin Secretion and Histological Appearance of Long-Standing Embryonic Pancreatic Grafts (A) Pig insulin levels following transplantation of E28, E42, E56, and E80 pig pancreas tissues under the kidney capsule of NOD-SCID mice. The data are based on average ± standard deviation pig insulin level measured in seven independent experiments, each of which includes comparison among pig pancreatic precursors of two to three different gestational ages (*, *p* < 0.05; ***, *p* < 0.005; comparing E42 or E56 with E28 pig insulin levels). (B) E42 pig pancreatic grafts 5 mo after transplantation under the kidney capsule of NOD-SCID mice. Macroscopic appearance reveals a large viable graft that covers the kidney and contains abundant blood vessels (panel A); the graft is marked by an arrow. Histological analysis of the grafts demonstrates mainly dense islets of different sizes (panel B) (Hematoxylin and Eosin staining; islets marked by arrows). The ability of these islets to produce hormones is evident by positive staining for insulin (panel C), glucagon (panel D), and pancreatic polypeptide (panel E). Close proximity between islets and ducts is occasionally seen (panel C, magnified inset). The epithelial cells are widely stained for cytokeratin 20 (panel F).

Taken together, our data show that an optimal gestational “window” is provided by the E42–E56 pig embryonic pancreas, exhibiting the most favorable potential to grow and differentiate into functional islets in NOD-SCID mice. However, this “window” could still be further defined by comparing the immunogenicity of E42 and E56 implants.

### Differential Immunogenicity of Pig Embryonic Tissue Obtained at the E42 and E56 Gestational Time Points

A major potential advantage of embryonic tissue implantation is its reduced immunogenicity [ 24, 25]. We demonstrated recently that marked differences in immune response are observed when pig or human embryonic kidney precursor tissues of different gestational ages are implanted in immunodeficient mice reconstituted with human lymphocytes [ 29]. Using the same approach, we tested the potential of the E42 and E56 pig pancreatic grafts to induce rejection by such “humanized” SCID mice.

In this model, engraftment of the hu-PBMCs in the NOD-SCID mice was documented by FACS analysis of human CD45-positive cells in the peritoneal fluid and by detection of human IgG in the serum of implanted mice using a human-IgG-specific ELISA (average IgG concentration: 60.7 ± 7.4 mcg/ml). Only mice positive for human IgG and for the presence of human CD3 cells were included. In each experiment, engraftment and growth of the fetal pig tissue implanted in NOD-SCID mice in the presence or absence of hu-PBMCs was monitored by ELISA for serum pig insulin levels. Figure 3 summarizes four experiments in which the pig insulin levels detected in the serum of grafted SCID mice were compared to those found in the grafted “humanized” SCID mice 4 wk after transplantation of E42 or E56 pig pancreatic tissue. As can be seen, in the absence of hu-PBMCs, similar pig insulin levels were found in NOD-SCID mice transplanted with E42 and E56 tissues (17.4 ± 7.2 and 21 ± 7.8 pmol/l, respectively). However, adoptive transfer of hu-PBMCs to the grafted NOD-SCID mice caused a significant reduction in the pig insulin level secreted by the E56 grafts relative to E56-grafted NOD-SCID mice in the absence of hu-PBMCs (5.76 ± 3 versus 21 ± 7.8 pmol/l, *p <* 0.001), whereas only a slight decrease in pig insulin levels was noted upon implantation of E42 pig pancreas in conjunction with hu-PBMCs (17.4 ± 7.2 versus 13.2 ± 5.4 pmol/l, *p* > 0.05). Similarly, the pig insulin level secreted by the E42 grafts in mice reconstituted with hu-PBMCs was significantly higher than that secreted by the E56 grafts implanted under similar conditions (13.2 ± 5.4 versus 5.76 ± 3 pmol/l, *p <* 0.001). The continuous insulin production in the E42 grafts in the presence of hu-PBMCs in contrast to the significant reduction of insulin secretion exhibited by E56 grafts indicates that indeed the earlier embryonic grafts are less immunogenic.

**Figure 3:**
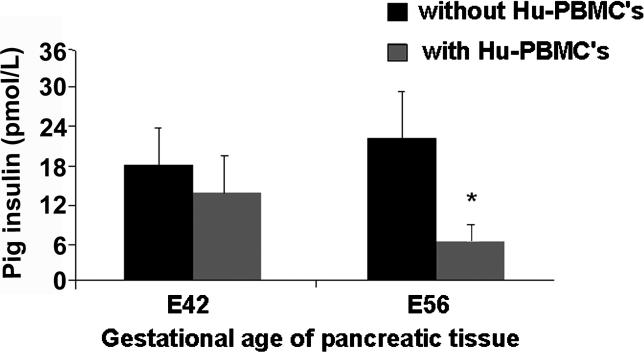
Insulin Secretion following Transplantation of E42 and E56 Pig Embryonic Pancreatic Tissues in the “Humanized” SCID Mouse Model Pig insulin levels were measured 4 wk after transplantation of E42 and E56 pig pancreas into NOD-SCID mice in the absence (black bars) and presence (grey bars) of 80 × 10 ^6^ adoptively transferred hu-PBMCs infused into the grafted mice 1–3 d after transplant. Four experiments were carried out, each comparing pig insulin levels secreted by the grafts from two gestational time points. The bars represent average ± standard deviation pig insulin levels measured in all experiments. The number of grafted NOD-SCID mice without and with hu-PBMCs, respectively, was as follows: 19 and 17 for E42, and 15 and 14 for E56.

The reduced immunogenicity exhibited by the early gestational age pig pancreatic tissue was further demonstrated by histological data specifically characterizing the subpopulations of the different inflammatory cells (panleukocytes, T and B lymphocytes, and macrophages) within the grafts. In contrast to the minimal hu-PBMC inflammatory cells observed within the E42 pig embryonic pancreatic grafts, an extensive hu-PBMC infiltrate was found within the E56 pig pancreatic grafts, leading to irreversible destruction of the graft (data not shown). Thus, although E42 and E56 pig pancreatic tissues exhibit similarly optimal growth potential, the less immunogenic E42 tissue likely represents the overall optimal pig embryonic source for transplantation.

### E42 Implants Are Predominantly Composed of Endocrine Tissue with Minimal Exocrine Activity

A major concern associated with pancreatic tissue implantation is how to avoid destruction of the growing organ by the local release of proteolytic enzymes, which might be released by the exocrine components of the implant. Therefore, we characterized the development and the proliferative potential of the endocrine and the exocrine embryonic pancreatic structures before and after transplantation of tissues harvested at various time points. Endocrine and exocrine elements were identified by immunostaining for insulin (as a marker for endocrine expression) or for trypsin and amylase (markers of exocrine expression); both types of cells were also stained for expression of Ki67 (a proliferation marker). When analyzing the embryonic tissues before transplantation (E28 to E80), the highest amount of insulin-containing islet-like structures, within which many cells coexpressed Ki67, was found in the E42 and E56 embryonic pancreas, indicating that at this stage a substantial population of cells could both proliferate and secrete insulin. At E80, the number of cells expressing both insulin and Ki67 markedly decreased, and multiple cells expressing trypsin and amylase were observed (data not shown).

Surprisingly, the relative expression of the exocrine and endocrine structures within the growing E42 grafts ( Figure 4) was entirely different from that observed in the adult pig pancreas, which is largely composed of exocrine tissue. As shown in Figure 4A, only a minimal level of exocrine cells was detected in the E42 graft 3 mo after transplantation, and most of the cells were of the endocrine lineage. Compared to the pretransplant E42 tissue, a decreased level of proliferating cells was noted, and the majority of the proliferating cells coexpressed insulin (arrows) and not trypsin or amylase. The disappearance of the exocrine tissue was even more pronounced at 6 mo post-transplant ( Figure 4B), at which time the graft was composed mainly of insulin-producing cells and exhibited a further decrease in proliferative potential (arrow). When evaluated in very long-term grafts at 8 mo ( Figure 4C) and 10 mo ( Figure 4D) after transplantation, no evidence of exocrine cells was found, and the grafts were completely occupied by enlarged islet structures not expressing proliferation markers.

**Figure 4:**
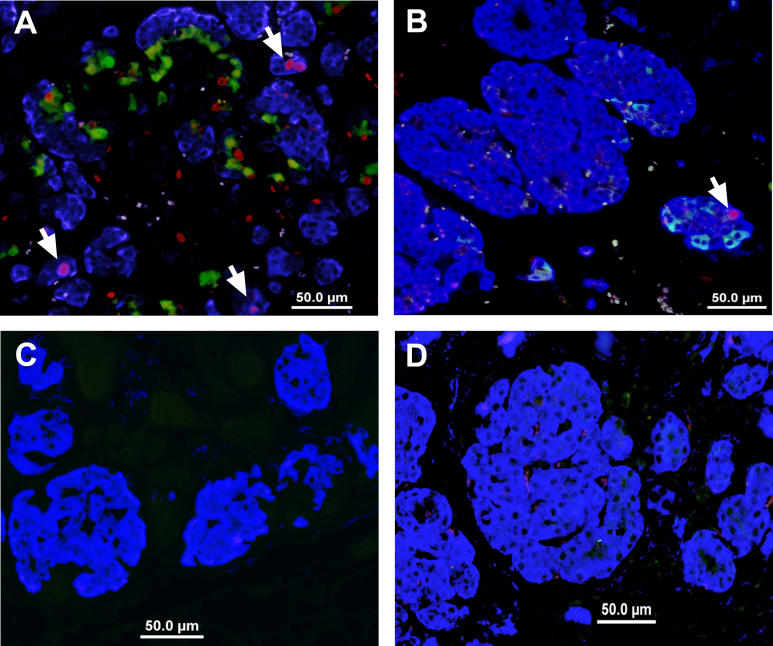
Expression of Endocrine, Exocrine, and Proliferative Markers in Long-Standing E42 Pig Pancreatic Grafts E42 pig pancreatic tissue was transplanted into NOD-SCID mice. Grafts were histologically analyzed at 3 (A), 6 (B), 8 (C), and 10 mo (D) after transplant. Endocrine expression is tracked by anti-insulin staining (blue), exocrine expression by anti-trypsin and anti-amylase staining (green), and proliferation status by anti-Ki67 staining (red). Cells coexpressing insulin and Ki67 are marked by arrows (A and B).

### Normalization of Blood Glucose Levels by E42 Pig Pancreatic Tissue

To determine the capacity of the pig embryonic pancreatic graft to control hyperglycemia in transplanted mice, we initially allowed the implanted tissue to grow and develop, and then we induced diabetes in the grafted NOD-SCID by alloxan [ 30]. This approach is based on the selective toxicity of alloxan, which destroys rodent, but not human or porcine beta cells, a circumstance that enabled us to assess the potential of the pig pancreatic implant to sustain normal glucose levels in a recipient whose own pancreas was practically destroyed [ 31, 32].

Experiments included 17 recipient mice grafted with growing E42 pig embryonic pancreas, 4 mo after transplant, followed for pig insulin level by ELISA, and 13 control non-grafted NOD-SCID mice. Both groups were intravenously injected with alloxan. Glucose concentrations were followed before and at various time points after alloxan injection. Prior to alloxan treatment, average glucose levels were similar in both grafted and control animals (5.6 ± 0.6 and 6.2 ± 1.1 mmol/l, respectively, *p* = 0.2). Pig insulin levels were negative in all control animals, while pig insulin levels in the E42-pancreas-grafted mice ranged from 18 to 1,086 pmol/l.

Within 3 d following alloxan injection, three animals in the control group had died, while the remaining ten survivors exhibited high glucose levels, with an average of 29.1 ± 5 mmol/l. The hyperglycemia persisted in all control mice, which eventually died within 18 d after alloxan injection ( Figure 5A). Of the 17 grafted mice, 13 did not develop hyperglycemia while four mice had glucose levels above 16.5 mmol/l after alloxan treatment. When analyzing the pig insulin level at 16 wk after transplantation (before alloxan injection), it was noted that the four mice who later failed to control hyperglycemia exhibited the lowest pig insulin levels (below 120 pmol/l), whereas in all 13 grafted mice exhibiting normal blood glucose levels following alloxan treatment the pig insulin level was above 120 pmol/l before alloxan treatment. To confirm the contribution of the graft to normalization of glucose levels in the euglycemic alloxan-treated grafted mice, grafts were removed by left nephrectomy at 41 d after alloxan injection in ten mice and at 61 d after alloxan injection in three mice, and blood glucose measurements were taken thereafter. As can be seen in Figure 5A, this intervention resulted in irreversible hyperglycemia.

**Figure 5:**
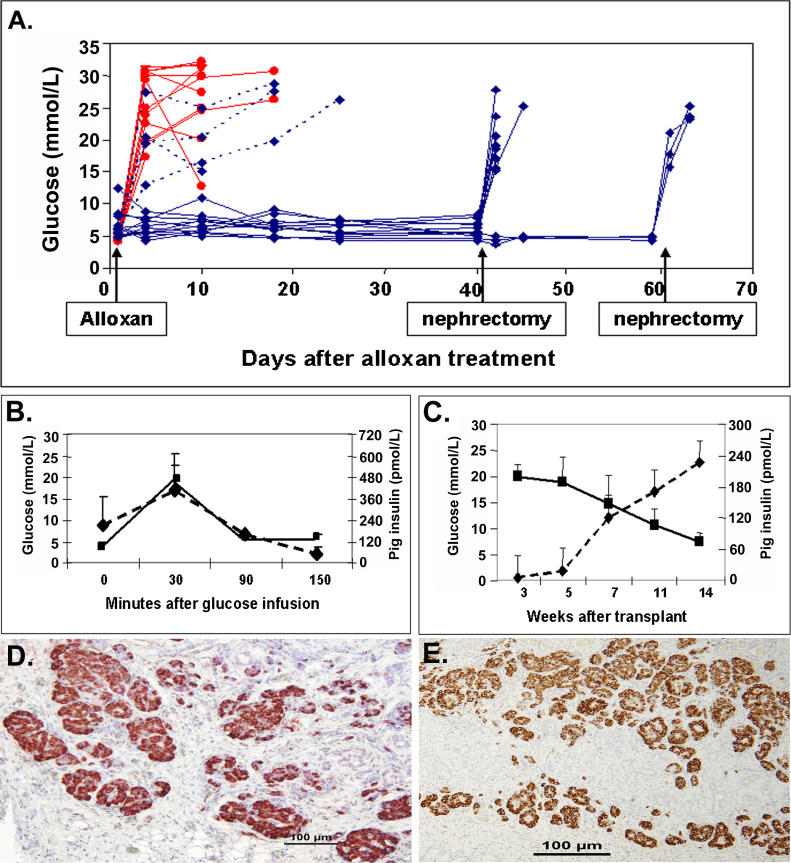
E42 Pig Pancreatic Tissue Normalizes Blood Glucose Levels in Diabetic Mice (A) Glucose levels in durably grafted (E42 embryonic pancreas) and non-grafted alloxan-treated NOD-SCID mice. Grafted (blue lines) and non-grafted (red lines) NOD-SCID mice were injected with alloxan 4 mo after transplantation. All non-grafted mice died within 2–18 d. Grafted mice exhibiting pig insulin levels below 120 pmol/l prior to the alloxan treatment failed to control hyperglycemia (broken blue lines); however, grafted mice demonstrating pig insulin levels above 120 pmol/l before alloxan injection maintained their glucose levels within the normal range (unbroken blue lines). Removal of the left kidney bearing the pig pancreatic graft at 41 or 61 d after alloxan treatment caused irreversible hyperglycemia. (B) Functionality of E42 pig pancreatic grafts in alloxan-treated NOD-SCID mice. After a 10-h fast period, 3 g/kg glucose was administered intraperitoneally. Glucose (black line) and pig insulin (broken black line) were followed at different time points spanning 150 min. The data represent three experiments and include 15 alloxan-treated NOD-SCID mice grafted with E42 pig pancreas evaluated 4 mo after transplant. (C) Long-term follow-up of average glucose (black line) and pig insulin (broken black line) in streptozotocin-treated NOD-SCID mice grafted with E42 pig pancreas. Of 19 animals treated with streptozotocin, ten survived up to 14 wk following transplantation and eventually became independent of exogenous insulin. (D and E) Pig insulin is highly expressed in the alloxan- (D) and the streptozotocin-treated (E) NOD-SCID mice 4 mo after transplantation, as detected by specific staining of nephrectomized kidneys bearing the E42 pancreatic grafts.

To assess the ability of the durable grafts to physiologically respond to changes in glucose levels, grafted alloxan-treated NOD-SCID mice were challenged with 3 g/kg glucose after fasting. As can be seen in Figure 5B, a physiological pattern of glucose levels and pig insulin secretion was found. The elevation in average glucose level 30 min after the glucose stimulation (4.3 ± 0.9 versus 22 ± 3 mmo/l, *p <* 0.001) was followed by insulin elevation (210 ± 120 versus 408 ± 132 pmol/l, *p <* 0.04), resulting in a gradual decrease of glucose level to a normal range, which was followed by insulin decrease.

The potential of E42 pig tissue to normalize blood glucose levels was also evaluated in NOD-SCID mice in which diabetes was induced by streptozotocin prior to implantation; this model resembles the clinical case when patients exhibit overt diabetes before transplant. In these mice, insulin treatment was required because of a glucose level above 13.8 mmol/l in 19 out of 19, 13 out of 15, nine out of 14, and two out of 12 surviving animals at 3, 5, 7, and 11 wk after transplantation, respectively, until the graft size was sufficiently large to secrete an average level of 228 ± 60 pmol/l pig insulin into the blood, a level usually achieved by 14 wk following transplantation. At this point, ten out of ten surviving grafted streptozotocin-treated mice were free of diabetes (highest glucose level: 9.1 mmol/l). Up to this point, as can be seen in Figure 5C, a gradual decrease in the glucose levels from 22.2 ± 2.3 mmol/l at 3 wk post-transplant down to 7.7 ± 1 mmol/l at 14 wk post transplant ( *p* < 0.001) was achieved. As in the alloxan model, nephrectomy of the kidney bearing the pancreatic graft was performed in five animals 3 wk after cessation of the exogenous insulin treatment, resulting in hyperglycemia (data not shown). Histology of the grafts within the nephrectomized kidneys in both alloxan- ( Figure 5D) and streptozotocin-induced ( Figure 5E) diabetes models revealed multiple islets embedded in fat and fibrous tissue, with no difference in their appearance and number per microscopic field.

### Implantation of E42 Pig Pancreatic Tissue into Fully Immune-Competent Recipients

Considering the low frequency of immature dendritic cells in peripheral blood mononuclear cell preparations, the “humanized” mouse model, which lacks mouse T cells or significant levels of mature dendritic cells or other professional human antigen-presenting cells (APCs), is biased towards measuring rejection mediated by the direct pathway in which T cells recognize foreign antigens presented on donor APCs [ 33, 34]. However, in fully immune-competent recipients, bearing normal levels of APCs as well as T cells, such grafts could still be rejected through the indirect pathway (cross-priming) in which host T cells recognize foreign antigens presented on host APCs. Indeed, when transplanted under the kidney capsule of fully immunocompetent mice in the absence of immune suppression, rejection of E42 pancreatic implants was completed between 8 and 10 d post-transplant, with fibrosis and infiltration observed at the implantation site, while implants in NOD-SCID recipients were fully viable ( Figure 6). Interestingly, all gestational age embryonic pancreas tissues (E28 to E100) were subjected to such prompt rejection when implanted in immune-competent mice. The rejection pattern of the E42 pig embryonic implants was further tested in mouse strains with various immune defects, including XID mice, in which B cell function is impaired, and nude mice, which are almost completely devoid of T lymphocytes. As seen in Figure 6C, complete rejection was detected in the XID mice, similar to the rejection pattern exhibited by normal C57BL/6 mice, while marked growth and development were found upon implantation of E42 tissue into nude mice ( Figure 6D). These results indicate that rejection of E42 pig pancreatic precursor tissue is largely mediated by T cells, and is less affected by humoral or natural killer responses.

**Figure 6:**
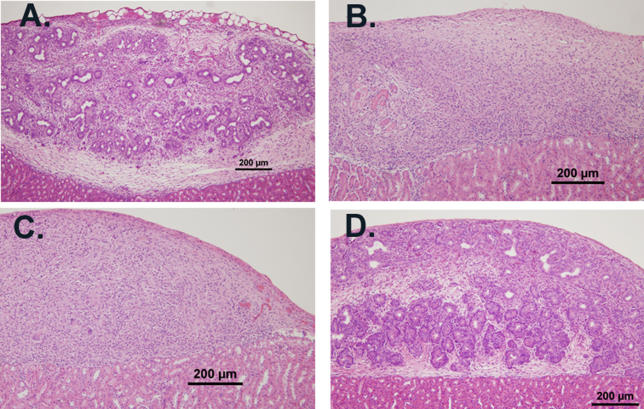
Rejection of the E42 Pig Pancreas in Different Immunologically Mutant Mice Grafts of E42 pig pancreas were transplanted under the kidney capsule of NOD-SCID (A), C57BL/6 (B), XID (C), and nude (D) mice and harvested 17 d after transplant. Note the extensive fibrosis and infiltration indicating rejection in the C57BL/6 and XID mice, while pancreatic components are seen in the NOD-SCID and nude mice. Each group included five mice.

The recognition that early embryonic kidney or pancreatic tissues are more likely to trigger cross-priming than direct recognition led us to investigate the role of costimulatory blockade, previously shown to be effective in the xenotransplantation model [ 35, 36]. To this end, we evaluated pancreatic implant growth and function after transplantation of E42, E56, and E80 pig pancreas into C57BL/6 mice in the presence of costimulatory blockade with CTLA4-Ig and anti-CD40L (administered on days 0, 2, 4, and 6 and thereafter every 2 wk, as described in detail in Methods). When tested at 2 wk post-transplant, the E80 grafts were completely rejected and the E56 grafts were heavily infiltrated, demonstrating advanced tissue damage (data not shown). In contrast, fully immune-competent recipients of E42 tissue, treated by biweekly administration of CTLA4-Ig and anti-CD40L, exhibited pig insulin blood levels similar to those found in NOD-SCID mice over the course of 3 mo after transplant ( Figure 7A). These results, which strongly indicate that rejection was avoided, were further confirmed by histological examination of the implants, which showed marked growth and development with minimal infiltration of mouse T cells or macrophages ( Figure 7B). Interestingly, cessation of treatment with the costimulatory agents resulted in reduced pig insulin levels and rejection of the implants (data not shown).

**Figure 7:**
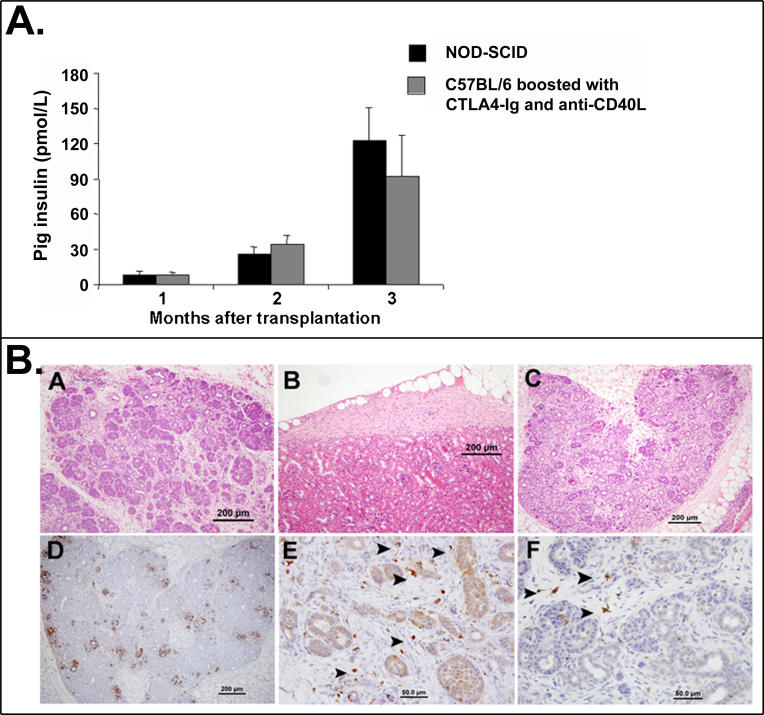
Pig Insulin Secretion and Histological Findings of E42 Pig Embryonic Pancreas Transplanted in NOD-SCID and Immunocompetent Mice Treated with Costimulatory Blockade (A) Pig insulin secretion by E42 pig pancreatic grafts implanted under the kidney capsule of NOD-SCID mice (black bars) or C57BL/6 mice treated every 2 wk with CTLA4-Ig and anti-CD40L (grey bars). No pig insulin could be detected in negative control transplanted C57BL/6 mice in the absence of immunosuppression, therefore these results are not shown. Each group included 13 mice. (B) Histological findings 3 mo following E42 pig pancreas transplantation under the kidney capsule of NOD-SCID mice (panel A), immunocompetent C57BL/6 mice (panel B), and C57BL/6 mice treated biweekly with CTAL4-Ig and anti-CD40L (panel C). Note the fierce rejection in C57BL/6 mice evident by implant destruction and fibrosis in (panel B), while intact graft development is demonstrated in C57BL/6 mice treated with CTLA4-Ig and anti-CD40L (panel C) revealing positive staining for insulin (panel D). A small number of mouse CD3 cells (panel E) and macrophages (panel F, stained by F4/80) infiltrated the graft parenchyma (marked by arrowheads) without causing apparent damage to the pancreatic structures.

Similarly to NOD-SCID recipients of E42 pancreatic tissue, when treated with alloxan 4 mo after transplantation, four out of seven grafted C57BL/6 mice treated biweekly for up to 4 mo post-transplant with CTAL4-Ig and anti-CD40L were able to sustain normal blood glucose levels, while all non-grafted alloxan-treated C57BL/6 mice exhibited irreversible hyperglycemia (8.5 ± 1.3 versus 31 ± 1.9 mmol/l, *p <* 0.001). Removal of the implants by nephrectomy in these four mice led to a sharp increase in average glucose levels 2 d after the surgical procedure (25.2 ± 1.3 mmol/l). Taken together, these results demonstrate that E42 pancreatic tissue can be functionally engrafted in fully immunocompetent mice for prolonged periods of time under an immune suppression protocol based solely on costimulatory blockade agents.

## Discussion

In the early 1990s, Groth et al. attempted to transplant embryonic pig pancreatic tissue into a series of diabetic patients [ 37]. In these attempts, which did not reverse diabetes, they used pooled porcine fetal islet-like cell clusters harvested between E66 and E81. Some evidence for porcine islet survival was detected in biopsies, but the insulin levels were not sufficient to maintain normoglycemia in human hosts. This outcome could be attributed either to rejection or to a variety of technical difficulties associated with the terminal condition of the patients, but the possibility that the embryonic tissue had weak growth potential because it was collected at a suboptimal gestation time should also be considered. Recently [ 27], we assessed in NOD-SCID mice the growth potential of several pig embryonic tissues obtained at different gestational time points. For liver precursors, optimal growth potential at 6 wk post-transplant was exhibited by E28 tissue, while pancreas and lung grew optimally if obtained at E42 and E56, respectively, with marked decrease in pancreatic growth potential around E80, the stage previously used for fetal islet-like cell cluster transplantation by Groth et al.

In the present study, our early pancreatic transplantation attempts have been extended over a long follow-up period, and the transplant “window” was assessed by functional as well as immunogenicity assays. Pig pancreatic tissue obtained at the E42–E56 gestational window led to the highest pig insulin blood levels in transplanted mice before and after glucose challenge 6 wk after transplantation. Furthermore, E42–E56 pancreatic tissue was found to secrete more pig insulin 4 mo after transplantation than E28 tissue, which has been previously advocated [ 38, 39], and E80 tissue, as was used for harvest of islet-like clusters in the human clinical trial [ 37, 40]. Considering that such clusters are less effective in general than entire tissue, likely because of the importance of stromal elements [ 41], the poor growth potential exhibited by E80 intact tissue fragments indicates that clusters isolated from E80 tissue will be even less effective.

The final choice between E42 and E56 was based on the reduced immunogenicity of E42 compared to E56 pancreatic tissue when transplanted in conjunction with human lymphocytes into NOD-SCID mice. It should be noted that adoptively transferred human lymphocytes tend to die of apoptosis by the second month post-transplant, and previous studies using this assay to monitor rejection of adult [ 42] or fetal tissues [ 26] have shown that optimal rejection is attained between 2 and 4 wk after infusion of hu-PBMCs. Thus, while significant reduction of insulin secretion accompanied by marked infiltration of human T or B cells, as well as macrophages, was found 4 wk after implantation of E56 tissue, only negligible peripheral infiltration of hu-PBMCs was observed in the growing E42 implant, and no significant reduction of insulin secretion could be detected.

Another major parameter in defining an ideal “window” for implantation of pig embryonic pancreas is the relative ratio of endocrine and exocrine elements exhibited by the growing tissue. Clearly, a minimal threshold of exocrine activity in the developing implant is critical for long-term preservation of the developing islets, which might be susceptible to destruction by proteolytic enzymes if the neighboring exocrine cells are predominant.

One approach to minimize the role of exocrine precursor tissue in the implant is to cultivate fetal islet-like cell clusters in short-term culture, as described by Korsgren et al. [ 43]. However, based on numerous studies demonstrating the active involvement of the surrounding mesenchyme in the pancreatic development and its role in endocrine cell differentiation and proliferation [ 41, 44– 50], we elected to transplant full embryonic pancreatic fragments. To minimize the pretransplant ischemia time, no preparative manipulations of the donor tissue were carried out, and fragments were transplanted immediately after harvest. Interestingly, prior to transplantation, exocrine elements could be detected only at stages beyond E56. Most importantly, the growing E42 tissue after transplantation exhibited a predominance of endocrine tissue, with only a minor exocrine component at 3 mo, and a complete dominance of islets without any trace of exocrine tissue by the sixth month post-transplant. The growing islets attained most of their size by the end of the fourth month post-transplant, though dividing cells (stained by Ki67) could be found up to the sixth month, when they reached their final size and stopped dividing, as indicated by examination at later time points. This plateau in growth and development is quantitatively measured by the insulin blood levels between 6 and 10 mo post-transplant. The intriguing predominance of endocrine tissue in the E42 implants might be related to low initial levels of exocrine precursor cells, but it may also be associated with unfavorable post-transplant microenvironmental conditions required for exocrine lineage development [ 51]. Interestingly, while second-trimester human pancreas (15–20 wk of gestation) transplantation into SCID mice showed similar diminished exocrine tissue in the grafts [ 52], the recent observation of Castaing et al. [ 53] that exocrine tissue is detected in early embryonic human pancreas grafts (7.5–9.5 wk of gestation) upon transplantation into SCID mice strongly indicates that the relative level of exocrine precursors or their ability to be induced by the stromal elements might vary between species.

The advantages of E42 porcine pancreatic tissue, as defined by its long-term growth potential, response to glucose challenge, reduced immunogenicity, and endocrine/exocrine ratio, were clearly exhibited when evaluating its curative potential in two different models of hyperglycemia. In the first model, irreversible pancreatic damage was induced by alloxan in long-term engrafted SCID mice. In the second model, streptozotocin-treated mice were treated by implantation of E42 tissue and treatment with exogenous insulin was maintained until the grafts were sufficiently large to sustain the mice in the absence of insulin treatment. In both models, a period of 3 mo of growth was required to effectively normalize glucose levels. Further studies are required to define whether the use of growth factors in vitro or in vivo might facilitate the growth of the embryonic implants so as to achieve normalization at an earlier time after implantation [ 54, 55].

While the results in SCID mice are encouraging, the immune barrier to xenografting, even when using early embryonic tissue, represents a major challenge. It has been known for over four decades that embryonic tissues are less immunogenic than their adult counterparts [ 56]. Accordingly, graft acceptance may reflect the progressive development of a complex array of cell surface molecules and soluble factors that determine immune recognition.

Clearly, because of the very low levels of human APCs in the SCID recipients, the assay of immune rejection by adoptively transferred hu-PBMCs employed in the present study is largely biased towards detection of the direct immune rejection mechanism and neglects the alternative indirect mechanism, in which donor antigens are presented by host APCs to host T cells (cross-priming). Therefore, although the E42 implant is less immunogenic and is relatively less susceptible to direct recognition than E56 tissue, it could still be rejected via the indirect pathway in other murine models. Indeed, when transplanted in fully immunocompetent mice, embryonic pig pancreatic tissues of all gestational ages were promptly rejected. The mechanisms underlying xenograft rejection of neovascularized embryonic tissues, such as pancreatic fragments, have not been extensively characterized to date. However, as shown in the present study by transplantation into mouse strains with various immunological defects, rejection is primarily dependent on T cell responses. Other studies describing xenorejection of later gestational age pig pancreas and adult pig islets also support this conclusion [ 57, 58]. In this context, it is likely that immunosuppressive agents, such as costimulatory blocking agents, directed against T cell activation and with minimal impact on angiogenesis and/or embryonic growth and development might be advantageous. Combined blockade of the CD28-B7 (by CTLA4-Ig) and CD40-CD154 (by anti-CD40L) costimulatory pathways has been shown to prevent anti-donor antibody production and is associated with increased host CD4+ T cell apoptosis in a concordant (rat to mouse) xenotransplantation model [ 59]. However, in our present study, when given during the first week after transplantation, the treatment protocol only delayed xenorejection of the E42 pancreas for about 1 mo. In contrast, repeated administration of CTLA4-Ig and anti-CD40L resulted in long-term graft survival with continuous production of insulin accompanied by normalization of glucose levels in diabetic mice. Thus, the pig embryonic tissue, which is markedly distinct from adult vascularized organs such as pig heart or kidney [ 60], might present immunological obstacles comparable to, but not more difficult than, those met with allotransplantation. Very recently, Hering et al. [ 61] showed that the immune suppression required to enable engraftment of adult pig islets in cynomolgus monkeys was rather toxic, leading to the death of six out of seven recipients that achieved normoglycemia but that did not survive because of the side effects associated with the immune suppression. Similar conclusions were also suggested by Cardona et al. [ 62]. The use of E42 pig tissue, which is less immunogenic than later gestational age tissues—not to mention adult tissue—might potentially enable the use of less toxic immune suppression protocols in the future.

The requirement for repeated treatment with costimulatory blockade agents suggests that permanent immune tolerance to the growing embryonic implants is not attained by this immunosuppressive treatment. However, the potential of these or other costimulatory blockade agents to yield permanent graft tolerance has not been fully investigated in the embryonic tissue model, and our current experiments are focused on defining an optimal combination of costimulatory blockade agents that might allow complete graft tolerance without continued medication. Also, considering the thrombotic activity recently exhibited by anti-CD40L antibodies in nonhuman primates and in humans [ 63, 64], it is important to test alternative costimulatory blockade agents, such as anti-LFA1, anti-ICOS1, or anti-CD48, which might effectively replace anti-CD40L in our model. Taken together, our data provide a proof of principle for the promising potential of E42 embryonic pig pancreatic tissue as a novel source for transplantation. Studies in a nonhuman primate model are warranted in order to investigate the scope of reduced immunogenicty, the issues of implant dose and transplantation site, and the functionality of the growing embryonic pancreas. If successful, the use of embryonic porcine tissues might offer an attractive source of unlimited pancreatic tissue.

## Abbreviations

APC: antigen-presenting cell
CD40L: CD40 ligand
E[number]: embryonic day [number]
hu-PBMC: human peripheral blood mononuclear cell
IgG: immunoglobin G
